# Editorial: Advancing health equity through surgery: a review of recent contributions

**DOI:** 10.3389/fsurg.2023.1292447

**Published:** 2023-09-21

**Authors:** Soham Bandyopadhyay

**Affiliations:** ^1^ Oxford University Global Surgery Group, Nuffield Department of Surgical Sciences, University of Oxford, Oxford, United Kingdom; ^2^ Clinical Neurosciences, Clinical & Experimental Sciences, Faculty of Medicine, University of Southampton, Southampton, Hampshire, United Kingdom; ^3^ Wessex Neurological Centre, University Hospital Southampton NHS Foundation Trust, Southampton, United Kingdom

**Keywords:** health equity, surgery, editorial, race & age & education & income & occupation, equality

**Editorial on the Research Topic** Advancing health equity through surgery: a review of recent contributions

The pursuit of health equity remains a moral imperative in modern surgical practice. The articles in this section contribute valuable insights into advancing health equity in various surgical disciplines. Each paper, whether explicitly or implicitly, serves to broaden our understanding of how surgical interventions can be both a tool and a metric for health equity.

## Clinical studies with equity implications

Xiaobin et al. present a prediction model for hepatic alveolar hydatid disease, an ailment that disproportionately affects marginalized communities. This model could be an essential tool for equitable healthcare resource allocation. Similarly, Nie et al. discuss pyogenic liver abscesses, emphasizing their experience in a traditional Chinese hospital, thereby spotlighting the need for culturally-sensitive healthcare approaches.

## Surgical techniques and health equity

Yao et al. explore the use of a trimodal prehabilitation model in lung cancer surgery. By tailoring preoperative care to patient-specific needs, they lay the groundwork for more equitable surgical outcomes. JinHua et al. examine a drainage technique for liver abscesses that could offer a more accessible treatment option for medically underserved populations.

## Racial and ethnoracial considerations

Wu et al. and Lv et al. directly confront issues of ethnoracial disparity in organ transplantation. Their work contributes vital data to our understanding of how racial and ethnic factors influence both waiting-list and post-transplant prognosis. These studies serve as an urgent call to address these disparities systemically.

## Age and comorbidity factors in health equity

Lv et al. delve into the often-overlooked subject of how age and metabolic syndrome affect treatment outcomes in prostate surgery, signaling the need for an equity-focused approach to comorbidity assessment. Bi et al. add to this discourse by investigating diastolic dysfunction in liver transplantation, highlighting a potential health equity consideration in transplant eligibility.

## Optimizing care for vulnerable populations

Zhou et al. investigate optimal oxycodone dosing for elderly patients undergoing gastrointestinal cancer surgery. By focusing on a vulnerable demographic, their work underscores the importance of age-sensitive care protocols in advancing health equity.

## Conclusion and future directions

The collective contributions in this section underscore the profound need for surgery to address health inequities. Future research must further delineate how surgical interventions can be optimized across diverse populations, thereby ensuring that the promise of health equity is not merely aspirational but actionable.

We look forward to additional submissions that explore the vast complexities of health equity in surgical practice, shedding light on the systemic changes required to ensure equitable surgical care for all.

By weaving these threads together, this section illustrates the multifaceted ways in which surgical research and practice can advance health equity. We invite future contributors to add to this crucial discourse.

